# Comparison of the Performance and Microbial Community Structure of Two Outdoor Pilot-Scale Photobioreactors Treating Digestate

**DOI:** 10.3390/microorganisms8111754

**Published:** 2020-11-08

**Authors:** Alessia Bani, Katia Parati, Anna Pozzi, Cristina Previtali, Graziella Bongioni, Andrea Pizzera, Elena Ficara, Micol Bellucci

**Affiliations:** 1 Istituto Sperimentale Lazzaro Spallanzani, Localita’ La Quercia, 26027 Rivolta d’Adda (CR), Italy; ab18858@essex.ac.uk (A.B.); katia.parati@istitutospallanzani.it (K.P.); anna.pozzi@istitutospallanzani.it (A.P.); cristina.previtali@istitutospallanzani.it (C.P.); graziella.bongioni@istitutospallanzani.it (G.B.); 2 Gruppo Ricicla labs., Dipartimento di Scienze Agrarie e Ambientali - Produzione, Territorio, Agroenergia (DiSAA), Università degli studi di Milano, Via Celoria 2, 20133 Milano, Italy; 3 Dipartimento di Ingegneria Civile e Ambientale (DICA), Politecnico di Milano, P.zza L. da Vinci 32, 20133 Milano, Italy; andrea.pizzera@mail.polimi.it (A.P.) elena.ficara@polimi.it (E.F.)

**Keywords:** microalgae-bacteria consortia, predation, raceway pond, bubble column reactors, nitrogen removal, productivity, centrate, molecular tools.

## Abstract

This study aimed at examining and comparing the nutrient removal efficiency, biomass productivity and microbial community structure of two outdoor pilot-scale photobioreactors, namely a bubble column and a raceway pond, treating the liquid fraction of an agricultural digestate. Bacterial and eukaryotic communities were characterized using a metabarcoding approach and quantitative PCR. The abundance, composition, diversity, and dynamics of the main microbes were then correlated to the environmental conditions and operational parameters of the reactors. Both photobioreactors were dominated either by *Chlorella* sp. or *Scenedesmus* sp. in function of temperature, irradiance and the nitrogen compounds derived by nitrification. Other species, such as *Chlamydomonas* and *Planktochlorella*, were sporadically present, demonstrating that they have more specific niche requirement. *Pseudomonas* sp. always dominated the bacterial community in both reactors, except in summertime, when a bloom of *Calothrix* occurred in the raceway pond. In autumn, the worsening of the climate conditions decreased the microalgal growth, promoting predation by *Vorticella* sp. The study highlights the factors influencing the structure and dynamics of the microbial consortia and which ecological mechanisms are driving the microbial shifts and the consequent reactor performance. On these bases, control strategies could be defined to optimize the management of the microalgal-based technologies.

## 1. Introduction

The water industry is facing a massive transition to transform wastewater treatment plants (WWTPs) into resource recovering factories [1]. In this light, the integration of microalgae-based technologies into WWTPs has gained attention because of their cost-effective bioremediation capability, the relatively low energy requirement, and the possibility to recover valuable biomass [2]. Microalgae-based processes have been successfully applied for the treatment and valorization of various municipal and industrial wastewaters, including secondary and tertiary effluents, piggery wastewater, digestate, and textile wastewater [3,4,5,6,7,8]. However, several drawbacks, including land requirement, uncompetitive harvesting and valorization costs, and a strict dependency on climatic and operational conditions still impede real implementation of algae-based wastewater treatment, especially in temperate zones [9].

Outdoor open systems (i.e., raceway ponds, bubble columns, flat panels) are considered so far as the most viable method of microalgal cultivation on wastewaters, though the treatment process is less controllable. Such systems are very susceptible to unavoidable environmental changes and contamination from the surrounding environments [10]. These conditions promote the development of complex and dynamic microbial consortia [11,12], which are composed not only of phototrophic organisms, but also of bacteria, protozoa, yeasts and fungi [13]. The community assembly, as well as the antagonistic and synergetic interactions within the organisms, competition for resources, and predation drive the system functions, namely nutrient removal rate and microalgal productivity. Therefore, understanding the biotic and abiotic mechanisms which favor the growth of desired microbial species or consortia might help to manipulate the community in a way to enhance treatment efficiency and productivity of valuable biomass. 

Correlations between the composition, diversity and distribution of mixed microalgae-bacteria consortia and biotic, environmental and operational parameters have been the focus of several recent studies [10,14,15]. Light and temperature are reported to control the growth of species belonging to Scenedesmacae or Chlorellacae within a mixed consortia [16], while nutrient depletion (i.e., N, P and CO_2_) plays a role in the accumulation of valuable compounds, such as oils and pigments, in the microalgal cells [17]. Grazers could destroy a microalgae cultivation system in a few days, causing important environmental and economic consequences [13,18]. The presence of active nitrifying bacteria (both ammonia-oxidizing bacteria (AOB) and nitrite-oxidizing bacteria (NOB)), which compete with the microalgae for ammonium, phosphorus, and CO_2_, might impact the microalgal biomass productivity [19,20]. In addition, nitrifiers oxidize ammonium, which might have an inhibitory effect, to nitrate, which is reported to be less easily assimilable than ammonium by the microalgae [21]. On the contrary, several bacteria are beneficial for the microalgal growth and seem to help in facing environmental perturbations and parasites. Bacteria could provide microalgae with CO_2_ through aerobic respiration, macro-micronutrients via nitrogen fixation and mineralization, but also synthetize compounds which stimulate various responses, such as the algal growth (vitamin B12), spore germination, pathogen resistance and cell aggregation [22]. Thanks to these research activities, several ecological mechanisms have been elucidated, with consequent improvements in the functioning and control of microalgae-based technologies. However, such systems are still not operated at their maximum capability and are subjected to unexpected failures. 

Further knowledge could be gained by fully characterizing the microbial community within the systems by advanced sequencing approaches. So far, the taxonomic identification of the dominant microalgal and zooplankton species was based on morphology through microscopic visualization [15,16], which is extremely time consuming and dependent on the operator’s skills. In addition, the characterization of the bacterial community structure in microalgal ponds is scarce and often performed with culturing-based techniques, which allow covering a limited portion of the biodiversity [23,24]. Amplicon-based metagenomics, also known as metabarcoding, targeting both eukaryotic and bacterial gene markers could provide deep insight into the complex community in the ponds and could help in elucidating the function and interaction between the different taxa. Such approach is still scarcely used in microalgal-based process [14,25,26,27], though frequently applied to unravel the microbial ecology mechanisms in engineered ecosystems treating waste and wastewater.

Given that, the goal of this study was to investigate how the structure and dynamics of the eukaryotic and bacterial communities in two types of outdoor photobioreactors, namely a raceway pond (RWP) and a column photobioreactor (PBR), treating the liquid fraction of centrifuged digestate, also known as centrate, change with seasonal and operational conditions and their impact on the photobioreactor functioning. A comprehensive characterization of the microbiome in the two reactors was assessed by a metabarcoding approach and compared. The composition, diversity and dynamics were then correlated with the operational parameters, meteorological conditions, and reactor performance (biomass productivity and nutrient removal rates). All data were then gathered to define the main drivers regulating the community assembly, as well as to provide useful information for the design, operation, and control of open real outdoor, open and non-sterile microalgal-based technologies. 

## 2. Materials and Methods 

### 2.1. Pilot Plant System and Operation

**Figure 1:**
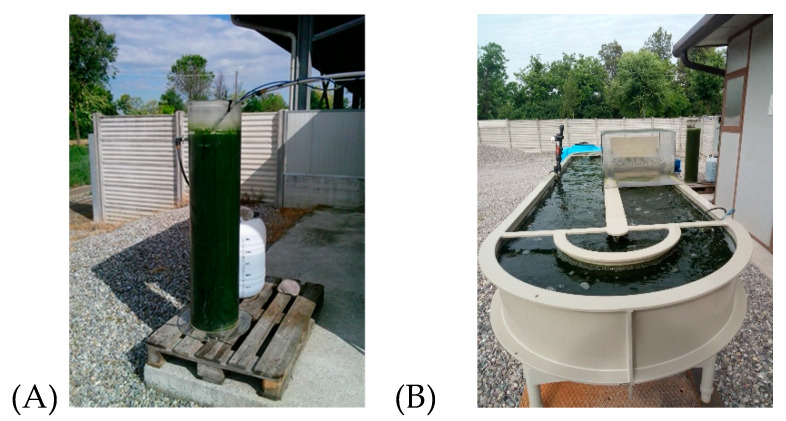
Bubble column photobioreactor (**A**) and a raceway pond (**B**).

The pilot plant for microalgae culturing was located in a piggery farm in the Cremona province (Northern Italy). The system consisted of two bioreactors operated in parallel, a bubble column photobioreactor (PBR) and a raceway pond (RWP), as described in Pizzera et al., 2019 [28]. The bioreactors where placed outdoors, facing the south in order to maximize sun exposure (Figure 1). 

Briefly, the RWP consisted of a main polypropylene tank 4 m long, 1 m wide, and 0.3 m deep (working volume = 880 L) with semi-circular edges and a contact cylinder for CO_2_ bubbling (named sump, height 0.8 m, diameter 0.44 m, volume 120 L). Mixing was ensured by a paddlewheel (20 rpm) and dissolved oxygen (DO) concentration, pH and temperature of the algal suspension were constantly monitored by two on-line probes connected to a control panel. CO_2_ was provided automatically from the bottom of the sump when the pH was above the set-point value of 7.5. The PBR consisted of a Plexiglass cylinder 131 cm high with an inner diameter of 29 cm (working volume of 75 L). Mixing and CO_2_ were provided by continuous air-bubbling (2.5 L h^−1^) through porous stones located at the bottom of the PBR. The pH was not controlled.

The PBR was initially inoculated with 8 L of undiluted centrate, 64 L of tap water and 0.5 L of a suspension of microalgae (2.5 × 10^6^ cell mL^−1^ of Chlorellaceae and 13 × 10^6^ cell mL^−1^ of Scenedesmaceae) previously grown on centrate from the same farm. After two months of operation, 65 L of algal suspension was transferred to the RWP and diluted in 90 L of centrate and 680 L of tap water to reach the final working volume. 

The centrate was derived from the full-scale anaerobic digester in the farm co-digesting piggery wastewater, energy crops and agricultural wastes. The main average characteristics of the centrate were: total Kjeldahl nitrogen (TKN) = 1290 mg L^−1^; NH_4_^+^-N = 1250 mg L^−1^; PO_4_^3−^-P = 54 mg L^−1^; soluble Chemical Oxygen Demand (COD) = 1520 mg L^−1^; total suspended solids (TSS) = 470 mg L^−1^. Both systems were fed on centrate, diluted with tap water (centrate:tap water, 1:5 *v*/*v*; after 143 days the RWP was fed on a centrate:tap water with a dilution of 1:3 *v*/*v*) with a hydraulic retention time (HRT) of 11 ± 4 days. The main characteristics of the diluted centrate feeding the two bioreactors are reported in Table 1. 

The functioning of the pilot plants had been monitored for 188 days, from May to November, when both reactors were in operation. Samples of the microalgal suspensions and centrate were collected ca. every two weeks from the PBR (for a total of 11 samples) and RWP (for a total of 12 samples) for physicochemical and biological analyses.

### 2.2. Analytical Methods

Filtered (0.45 μm) samples of the microalgal suspensions (10 mL) were used for the determination of the concentration of ammonium, nitrate, nitrite, orthophosphate phosphorus (PO_4_-P) and soluble COD by spectrophotometric test kits (Hach-Lange, DR6000TM UV VIS Spectrophotometer, Hach Lange LT200 Dry thermostat). Total and volatile suspended solids (TSS and VSS) were determined in duplicate according to Standard Methods (APHA, 2005). The pH was measured by a portable pH meter (Hach-Lange HQ40d) in the PBR and with the on-line probe in the RWP. Absorbance at 680 nm (OD_680_) and turbidity were measured spectrophotometrically (Hach-Lange, DR6000TM UV VIS Spectrophotometer) as described elsewhere [28]. Irradiation and temperature data were obtained from the website of Arpa Lombardia.

### 2.3. Operational Data Processing

The performance of the reactors was monitored by computing the volumetric production (*pr*, in mg L^−1^ d^−1^) of each relevant component (C_j_ with j = VSS, COD, N-forms, P) from the dynamic mass balance, which considers both the PBR and the RWP as completely stirred tank reactors, as follows:

(1) $$pr_{j,ti}=\frac{Cj_{OUT,t_{i}}-Cj_{OUT,t_{i-1}}}{t_{i}-t_{i-1}}+\frac{\left(\frac{Cj_{OUT,t_{i}}+Cj_{OUT,t_{i-1}}}{2}\right)}{HRT}\;-\frac{\frac{Cj_{IN,t_{i}}+Cj_{IN,t_{i-1}}}{2}}{HRT}$$

where C_IN_ and C_OUT_ are the concentration of the component in the feeding diluted centrate and in the algal suspension, at two consecutive sampling dates (time t_i−1_ and t_i_), and HRT is the average hydraulic retention time in the period, respectively.

Negative results indicate that the component is removed, therefore it is reported as removal rate (*rr*). This is valid for COD and ammoniacal nitrogen. The ammonium oxidation rate (*pr*N-NO_x_) was calculated as the sum of the produced oxidized nitrogen forms (*pr*NO_2_^−^-N+ *pr*NO_3_^−^-N). 

Finally, removal efficiencies were computed for each relevant contaminant (Cj) as follows:

(2) $$\eta_{j,ti}=\frac{C_{j,INt_{i}}-C_{j,OUT,t_{i}}}{C_{j,INt_{i}}}\times 100$$

Free ammonia (mg/L) was computed by considering the concentration of the total ammoniacal nitrogen (TAN), temperature and pH, as described elsewhere [29]. 

### 2.4. DNA Extraction and Sequencing

For each sampling event, 2 mL of bulk suspensions of each photobioreactor were collected for DNA extraction. The samples were snap frozen in liquid nitrogen at the sampling location and then transported to the laboratory where they were processed. The material was lyophilized in a Christ Alpha 1-4 lsc lyophilizer at −20 °C and 0.520 mbar pressure. DNA was extracted with DNeasy Plant Kit (Qiagen, Milan Italy) according to the user’s manual. The resulting DNA was quantified with Qubit Fluorometer (Invitrogen, Monza, Italy) and sent to the IGATech sequencing center (IGA Technology Services s.r.l., Udine, Italy) to perform Illumina sequencing on the 16S rDNA gene for bacteria and the internal transcribed spacer (ITS) of nuclear DNA for eukaryotes. In particular, the V3-V4 region was chosen for the 16S rDNA gene (341F 5′-CCTACGGGNGGCWGCAG-3′; 805R 5′-GACTACHVGGGTATCTAATCC-3′ [30], while the ITS region (ITS1 5′-TCCGTAGGTGAACCTGCGG-3′; ITS4 5′-TCCTCCGCTTATTGATATGC -3′, [31]) was used for the eukaryotes.

### 2.5. Bacterial Quantification by Real-Time Polymerase Chain Reaction 

The total bacteria and ammonia oxidizing bacteria (AOB) were quantified by real time or quantitative PCR (qPCR) using primer set by targeting the 16S rRNA (1055f 5′-ATGGCTGTCGTCAGCT-3′; 1392r 5′-ACGGGCGGTGTGTAC-3′ [32]) and AmoA (AmoA1F 5′-GGGGTTTCTACTGGTGGT-3′; AmoA2R 5′-CCCCTCKGSAAAGCCTTCTTC-3′ [33]) genes, respectively. Each PCR reaction (20 µL final volume) contained 1 × PowerUp SYBR Green Master Mix (Applied Biosystem, Monza, Italy) (10 µL), forward and reverse primers (100 nM each primer, see below), 0.4 mg mL^−1^ bovine serum albumine (BSA, Thermo Fisher, Monza, Italy), distilled water (RNase/DNase free, Thermo Fisher, Monza, Italy) and 2 µL of DNA (ten-fold diluted). The reactions were performed on a 7500 Fast Real-Time PCR system (Applied Biosystem, Monza, Italy) equipped with Applied Biosystems 7500/7500 Fast Real-Time PCR Software (7500/7500 Fast software). PCR reactions were performed as described in Marazzi et al., 2019 [34]. Standard DNA were constructed from purified PCR amplicons from pure cultures of *Nitrosomonas communis* (DSMZ 2843) for bacterial, and *Nitrosomonas eutropha* (DSMZ 101675) for AOB. All standards consisted of ten-fold dilutions ranging from 10^8^ to 10^1^ copies µL^−1^, and DNA samples were run in triplicate using the following cycling conditions: 95 °C denaturation for 10 min, followed by 40 cycles of 20 s at 95 °C, 15 s at 58 °C and 30 s at 72 °C (as described in Bani et al., 2019 [35]) and 95 °C for 2 min denaturation followed by 40 cycles of 45 s at 94 °C, 30 s at 56 °C and 60 s at 72 °C (as described by Bellucci et al., 2015, [36]). To check the amplicon quality and potential primer dimer formation, PCR runs were completed with a melting analysis starting from 65 to 95 °C with temperature increments of 0.25 °C and a transition rate of 5 s. The total number of bacteria was estimated from the 16S rRNA gene copy numbers, by assuming that an average of 4.2 rRNA operon exists per cell [37]. The *AmoA* gene copy number was converted in AOB cell number by assuming 2 copies of the gene in each cell [38]. The cell-specific ammonium oxidation rates (CSAOR) of the AOB were calculated by taking into consideration the amount of ammonium oxidized and the number of AOB as previously reported [39,40].

### 2.6. Bioinformatics

Amplicon sequences were analyzed as follows. Reads were demultiplexed based on Illumina indexing. For 16S sequences, reads were overlapped and the not overlapping discarded. Low quality sequences and sequences shorter than 200 bp were removed. QIIME open-source pipelines [41] were used for the following steps: chimera check, grouping replicate sequences and picking the operational taxonomic units (OTU). The OTU definition was based on the USEARCH algorithm [42] by setting a 97% similarity threshold. Taxonomy assignation was done on the classifier tool of the Ribosomal Database Project (RDP) with the default setting [43]. For the ITS sequences, a different approach was used due to the non-overlapping nature of reads. All sequences were filtered based on the quality and length of the reads (longer than 200 bp). Then, the same steps previously described for the 16S rRNA amplicon were applied, except for the taxonomic classification, which was performed by comparing the sequences with those presents on the National Center for Biotechnology Information (NCBI) database. Sequences were submitted in the NCBI SRA data archive under the code PRJNA634916.

### 2.7. Statistical Analysis

Statistical analyses were performed using available packages of R (version 3.6.0) [44]. Paired sample *t*-tests were carried out to compare the performance and efficiency between reactors, as well as the total and nitrifying bacterial abundances in the two systems. Matrices based on Pearson correlation were built to find correlations among physicochemical data and operational parameters of the reactors (temperature (Tr), pH, dissolved oxygen; inlet and outlet COD, NH_4_^+^-N, NO_2_^-^-N, NO_3_^-^-N, PO_4_, TSS, VSS, turbidity; N:P ratio in the influent; free ammonia (FA) in the microalgal suspensions; HRT), climatic conditions (averaged daily irradiance and temperature), reactor performance (*rr*COD, *rr*TAN, *pr*NO_x_-N, *pr*NO_2_^-^-N, *pr*NO_3_^-^-N, *rr*PO4, *pr*VSS), and microbiological data (microalgal density (OD_680_), numbers of the total and nitrifying bacteria, species richness of the total bacteria, eukaryotes and microalgae). 

The vegan package was used [45] for creating rarefaction curves to test the depth of the sequencing. Species richness of the bacterial (N_BAC_) and eukaryotic (N_EK_) communities were estimated by considering each OTU as a singular microbial species. As in Borruso et al., 2018 [46], the number of shared eukaryotic OTUs among PBR and RWP was calculated by considering only the OTUs with a relative abundance higher than 1%. Microalgal species richness was correlated with the biomass productivity, considered as *pr*VSS, and stability, estimated by the standard deviation of three consecutive *pr*VSS values (same sampling day, previous and successive sampling days).

Non-metric multidimensional scaling (NMDS) plots, combined with the function *envfit* of vegan, were used as preliminary assays to elucidate how the bacterial and eukaryotic communities were influenced by the physicochemical and operational parameters, and climatic conditions. The analyses were conducted by using the bacterial and eukaryotic patterns and the operational parameters and climatic conditions detected at the same sampling time.

Clustering analyses based on the Bray–Curtis similarity index, combined with SIMPROF to check for significance (*p*-value < 0.05), were performed with the package *clustsig* [47] in order to determine significant changes in the main eukaryotic (species level, threshold 1%) and bacterial (genus level, threshold 10%) populations. The heatmaps were built with the same pool of data using the package *ggplot2* [48].

To elucidate which abiotic factors influenced the shape and evolution of the microbial communities, the relative abundance of the dominant eukaryotic (species level, threshold 1%) and bacteria (genus level, threshold 10%) was correlated to the mean of the physicochemical data, operational parameters and climatic conditions, which were detected over the 7 days preceding the sampling time. In this case, the Kendall rank correlation coefficient was used, and matrices were built with the package *corrplot* [49]. Only correlations with a *p*-value lower than 0.05 are reported.

OTUs with a relative abundance higher than 1% were also used for bipartite networks, which were created using *CoNet* function [50] inside the package *Cytoscape* [51]. Different correlation coefficients were used: Pearson and Spearman, and dissimilarity indices, Bray-Curtis and Kullback-Leibler, permutation and bootstrap score were based on 1000 interactions. All major network indices were calculated using the *igraph* package [52].

## 3. Results

### 3.1. Performance of the Bioreactors

**Figure 2:**
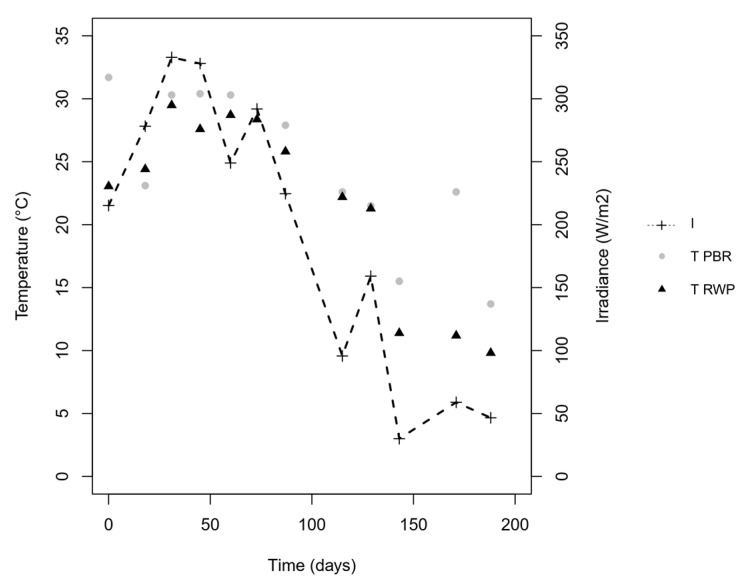
Temperature detected inside the PBR (grey) and RWP (black) and irradiance (dashed line).

Irradiance and temperature values detected during the sampling campaign are reported in Figure 2.

**Figure 3:**
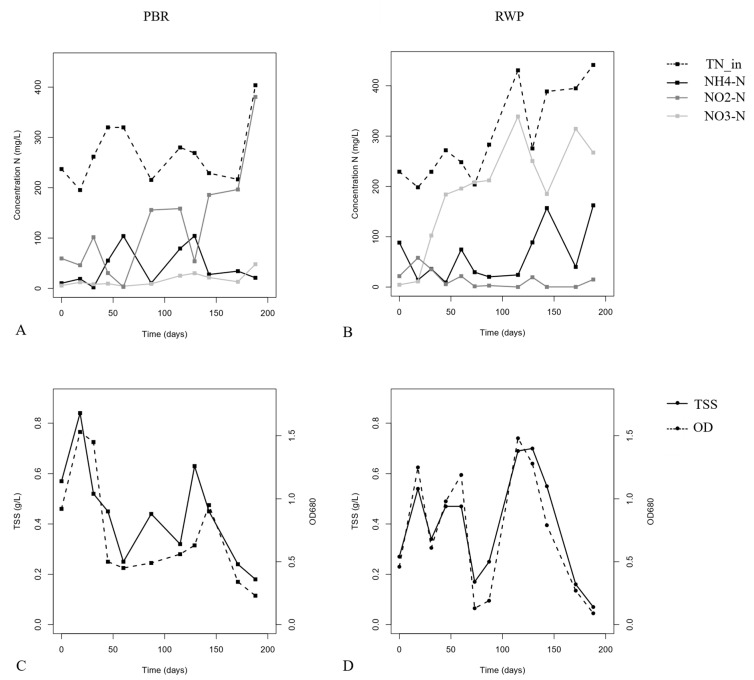
Concentration of the total nitrogen (TN) in the influent (dashed line), ammonium (solid black line), nitrite (solid dark grey line) and nitrate (solid light grey line) measured in PBR (**A**) and RWP (**B**). Microalgal density measured as optical density (OD_680_) (dashed line) and total suspended solid (TSS) concentration (solid line) detected over time into PBR (**C**) and RWP (**D**).

The irradiance and temperature figures, which were high at the beginning of the trial (end of May), with peak at the end of June and July (day 31 and 45), and low in September (day 115), are typical of the continental temperate climate of the Padan Plain. The concentrations of ammonium, nitrate, and nitrite measured in the two reactors are shown in Figure 3A,B, while Table 2 summarizes the mean physicochemical parameters measured in the reactors, as well as the nutrient and COD removal and production rates. 

The average pH in RWP was 7.2 ± 0.6, while the average pH in PBR was 8.5 ± 0.5, because the pH was not controlled by the addition of CO_2_ (*p*-value < 0.05, paired t-test). The ammoniacal nitrogen removal rates and efficiencies were similar in both reactors, averaging 20.6 ± 6.8 mg L^−1^ d^−1^ and 81.5% ± 14% *(p*-values > 0.1, t-paired test), respectively, indicating that the diverse configurations of the reactors did not impact on the overall NH_4_^+^-N removal. Most of the ammonium was oxidized (*pr*NO_x_-N = 16 ± 9 mg L^−1^ d^−1^) through nitrification. However, the nitrate production rate in RWP was much higher than in PBR (*pr*NO_3_^-^-N = 1 ± 1 mg L^−1^ d^−1^ in the PBR and 16.7 ± 7.9 mg L^−1^ d^−1^ in the RWP), indicating that complete oxidation of ammonium to nitrate could be achieved only in the RWP, while partial nitrification to nitrite occurred in the PBR. No significant differences in the residual concentration of COD (Table 2) between reactors were found and the *rr*COD was negligible.

### 3.2. Biomass Productivity and Abundance of the Microbial Populations

The microalgal density, which was measured as OD_680_, varied between 0.09 and 1.53, and the trend over time concurred with the concentrations of the TSS as shown in Figure 3C,D (r = 0.87, *p*-value < 0.01). The biomass productivity fluctuated, but the average value (25 ± 20 mg VSS L^−1^ d^−1^) was similar in both configurations (*p*-value = 0.26, paired *t*-test) and comparable with the range reported in the literature for microalgae culturing on the liquid fraction of digestate [8].

**Figure 4:**
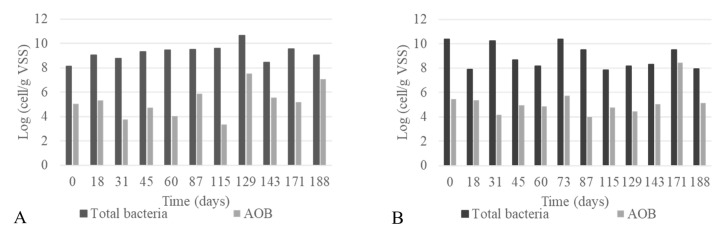
Concentration of the total (dark grey) and ammonia oxidizing (light grey) bacteria detected over time into the PBR (**A**) and the RWP (**B**).

Figure 4A,B show that the total bacterial concentration ranged between 4.4 × 10^10^ and 2.8 × 10^8^ cell g VSS^−1^_,_ significant differences between reactors (*p*-value > 0.1, paired *t*-test). However, a significant negative correlation between the bacterial number and microalgal density was observed (r = −0.72, *p*-value = 0.0001). The numbers of AOB, which were always two or three orders of magnitude lower than the total bacteria, were similar in the two reactors (*p*-value > 0.05, paired *t*-test), justifying the comparable ammonium oxidation rate observed. Nevertheless, the cell-specific ammonium oxidation rates (CSAOR, logCSAOR ranging between −0.2 and 5 fmol cell^−1^ h^−1^) were significantly higher than the ones reported for these types of bacteria [53], suggesting that the detected number of AOB was underestimated or other microorganisms contributed to the process.

### 3.3. Composition of the Eukaryotic and Bacterial Communities 

At the phylum level, the eukaryotic communities of both reactors were dominated by microorganism belonging to Chlorophyta (58% ± 38% in PBR and 63% ± 33% in RWP), Ciliophora (23% ± 38% in PBR and 26% ± 29% in RWP), and Fungi (5% ±15% in PBR). Chlorophyta are commonly found in outdoor cultivation systems fed on centrate because of their resistance to harsh cultivation conditions [54,55], while members of Ciliophora are algivorous and microalgal grazers, frequently reported in microalgal biomass culturing systems [13,18]. Their high relative abundance, the percentage of which is also negatively correlated with the microalgal one (r = −0.9, *p*-value < 0.001), is an indication of intense predation. 

**Figure 5:**
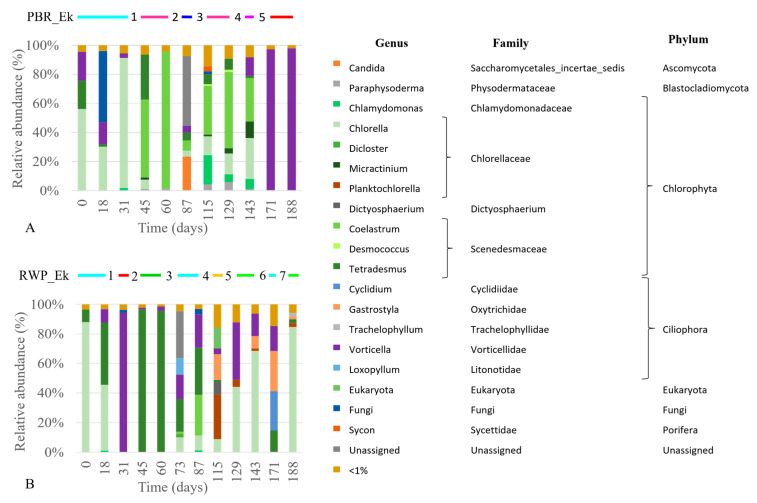
Relative abundance of the main eukaryotic organisms (genus level higher than 1%) detected in the PBR (**A**) and RWP (**B**). Taxonomic ranks (Family and Phylum) are also provided. Above the bar plots, significant population shifts, which were assessed by the combined SIMPROF and clustering analyses, are reported. For line colors refer to the dendrogram in Figure 7A, while the numbers identified the codes in Table 4.

The dominant eukaryotic genera detected by the metabarcoding approach are reported in Figure 5, while in Figure S1 the main microalgae and Ciliophora identified by the optical microscope are shown.

In the PBR, the main microalgal genera were *Chlorella* (23%± 29%) and *Coelastrum* (26% ± 33%) (Figure 5A). *Chlorella* was dominant until day 45, when it was replaced by *Coelastrum*. *Vorticella* contributed to the 6.1±7.7% of the total eukaryotic community most of the time, but it was the sole genus detected in the last two samples. Differently, in the RWP (Figure 5B) the microalgal community was dominated by members belonging to *Chlorella* (31% ± 36%) and *Tetradesmus* (27% ± 36%). *Chlorella* was replaced by *Tetradesmus* between days 45 and 73, when *Chlorella* returned to be the dominant genus. Members closely related to the *Coelastrum* genus were barely detected in the RWP (3% ± 8%), while a severe bloom of *Vorticella* sp. occurred at day 31, when the system faced a drastic reduction in the microalgal density (Figure 3D).

**Figure 6:**
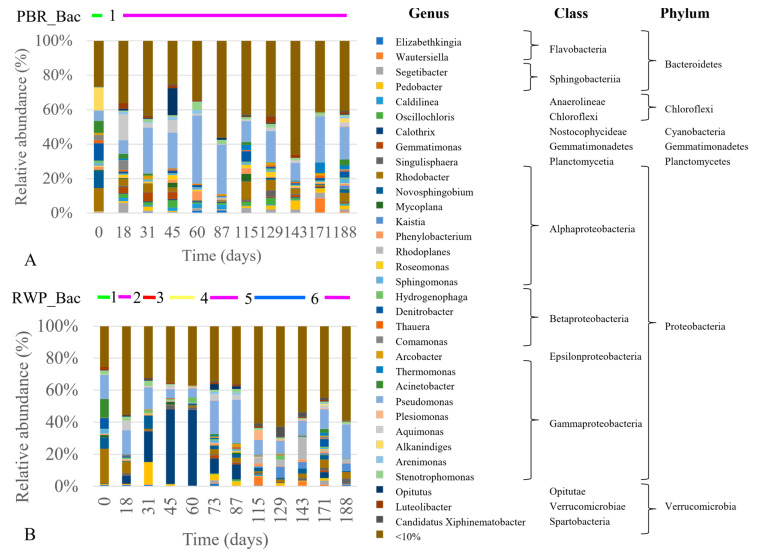
Relative abundance of the main bacterial genera (>10%) detected in the RWP (**A**) and PBR (**B**). Taxonomic ranks (Class and Phylum) are also provided. Above the bar plots, significant shifts of the bacterial communities, assessed by the combined SIMPROF and clustering analyses, are reported. For line colors refer to the dendrogram in Figure 7B, while the numbers identified the codes in Table 4.

As for the bacterial community (Figure 6A,B), at phylum level, Proteobacteria were dominant in both reactors (59% ± 14% and 49% ± 16% in PBR and RWP respectively), followed by Cyanobacteria (13% ± 11% PBR and 28% ± 15% RWP), and Bacteroidetes (10% ± 5% in PBR and 13% ± 6% in the RWP). Proteobacteria and Bacteroidetes have been already acknowledged to be dominant in high-rate algae ponds [14], as well as in the phycosphere of freshwater microalgal strains [56], while cyanobacteria, being phototrophic, co-exist commonly with microalgae in photobioreactors treating wastewater [10,14]. As for class composition, the PBR was dominated by Gammaproteobacteria (31% ± 9%), followed by Alphaproteobacteria (16% ± 9%) and Nostocophycideae (13% ± 11%), while in the RWP, dominance of Nostocophycideae (26% ± 14%), Gammaproteobacteria (21% ± 9%) and Alphaproteobacteria (18% ± 10%) was revealed. At the genus level, members belonging to *Pseudomonas* were the most abundant in both reactors (20% ± 10% and 14% ± 7%). Their high number is not surprising, as *Pseudomonas* spp. are ubiquitous in freshwater and soil.

Other common genera found in the two photobioreactors were: *Rhodobacter* (4% ± 4% and 4% ± 6%), *Pedobacter* (1% ± 2% and 2% ± 4%), *Opitutus* (2% ± 5% and 0.4% ± 1%). For PBR, the most common genera were instead *Aquimonas* (3% ± 4%), *Denitrobacter* (2% ± 3%), *Oscillochloris* (2% ± 2%), *Segetibacter* (2% ± 2%) and *Stenotrophomonas* (1.5% ± 1%). In RWP, *Calothrix* (12% ±1 8%) became the dominant genera at days 45 and 60. Other specific genera found in the RWP were: *Kaistia* (2% ±3%), *Novosphingobium* (2% ±3%), *Rhodoplanes* (2% ±4%).

### 3.4. Diversity of the Eukaryotic and Bacterial Communities

The total species richness (N) of the bacteria (N_BAC_ = 3224 ± 1589) was higher than the eukaryotic one (N_EK_ = 574 ± 442) (Figure S2). Yet, a more diverse bacterial community was detected in PBR than in RWP (*p*-value = 0.02; paired-*t* test), while no differences in N_EK_ could be observed between reactors. Both bacterial and eukaryotic species numbers varied over time. The highest values of N_EK_ concurred with the mitigation of the irradiation and temperature conditions after days 87, suggesting that a low to intermediate amount of resources (i.e. irradiance) fosters diversity [57]. A more diverse community in a reactor should increase the productivity and functional stability, especially system resilience to perturbations. However, eukaryotes also include the microalgae predators, which perturbed the ecosystem. Therefore, to better understand the diversity–ecosystem function relationship in the reactors, microalgal and non-microalgal species richness should be differentiated.

The total number of Eukaryotic species with a relative abundance higher than 1% is reported in Table 3. A total of 63 species could be detected, 40 of which were not associated with microalgae, indicating that the reactors were colonized by diverse protozoa, fungi and yeast. Only two species (*Vorticella microstoma* and an undefined fungus) could be found in both reactors, while 11 and 27 species could be detected only in the PBR and RWP, respectively. The higher diversity of non-microalgal eukaryotes found in the RWP than in the PBR indicates that the raceway was more prone to contamination. Besides having a much more open surface than the PBR, sudden variations in the operational parameters, such as the sharp increase of TAN in the influent (between day 87 and 115) and changes in the dilution rate (between day 143 and 171), stressed the microalgal community favoring the growth of grazers and predators. 

As for microalgae, a total of 23 species could be observed; 34.8% (8 out of 23) of them were shared among reactors. Species belonging to the genera of *Chlamydomonas*, *Chlorella*, *Tetradesmus* and *Coelastrum* were detected in both systems demonstrating their high versatility, while *Desmococcus*, *Micractinium, Dicloster*, *Planktonchlorella* and *Dictyosphaerum* were present either in the PBR or RWP, suggesting that they have more definite habitats.

### 3.5. Associations between the Microbial Community Structure and the Abiotic and Biotic Parameters

**Figure 7:**
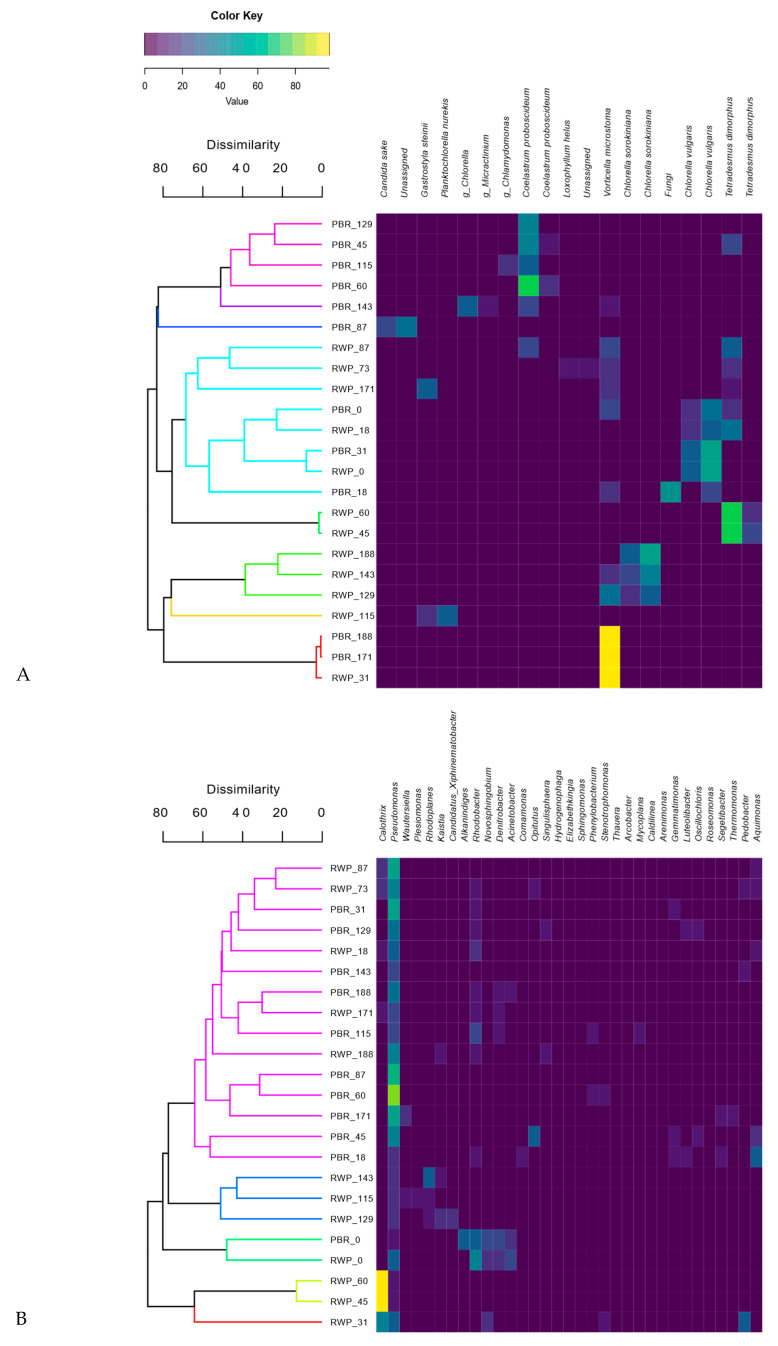
Heatmap and dendrogram based on Bray-Curtis similarity matrices identifying the main significant eukaryotic (species level) (**A**) and bacterial (genera level) (**B**) population changes detected by SIMPROF analyses. The colored clusters in the dendrogram are representative of statistically similar community patterns.

Clustering analyses show that the bacterial and eukaryotic patterns had grouped mostly according to the reactor configuration (Figure 7A,B).

This, together with the results of the NMDS analyses (Figure S3), indicates that the microbial community assembly was influenced not only by the meteorological conditions, but also by the geometry of the reactors, their different operational parameters and nitrification pathways. Indeed, the main factors affecting the eukaryotic community composition were irradiance, temperature, and the level of N compounds in the inlet and inside the reactors, whereas the bacterial community composition was mostly influenced by P, irradiance, temperature and level of TAN in the influent.

**Figure 8:**
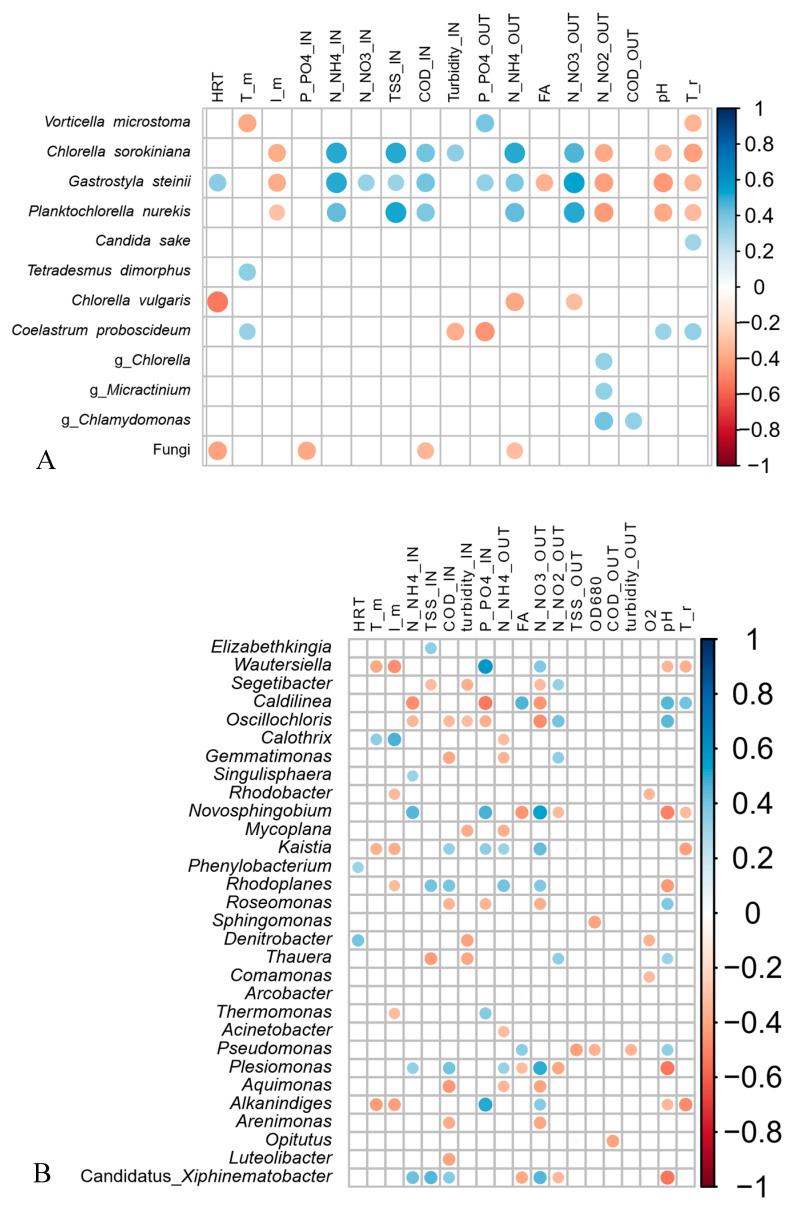
Correlation patterns between the relative abundance of the main eukaryotic (species level, threshold 1%) (**A**) and bacterial (genus level, threshold 10%) (**B**) populations, which were detected in the two reactors, and the mean of the physicochemical and operational parameters measured in the influent (IN) and in the photobioreactors (OUT) the previous seven days based on the Kendall rank correlation coefficient. Only significant positive (blue) and negative (red) correlations are shown (*p*-value < 0.05) in the matrices. I_m and T_m are the average daily values of the solar irradiance and temperature, respectively; T_r is the temperature detected in the reactors, FA is the free ammonia and O_2_ is the dissolved oxygen concentration.

Nevertheless, several samples collected in the same reactor but at different times were found in statistically different groups, demonstrating significant changes in the communities. The eukaryotic community shifted five and seven times in PBR and RWP, respectively, while one significant change was observed in the bacterial community in PBR and six in the RWP. To establish which factors favored or inhibited the growth of the dominant bacteria, microalgae and predators in each group, giving rise to significant population changes or system failures, the relative abundance of the main microorganisms were evaluated for their correlation with the main operational parameters and meteorological conditions (Figure 8). As regards microalgae, shifts between Chlorellaceae and Scenedesmaceae in the reactors depended on the temperature, irradiance, concentration of solids in the centrate, which in turn affects light penetration, pH and concentration of N forms. High irradiance and T promoted the Scenedesmaceae (i.e., *Tetradesmus* and *Coleastrum*), which are more tolerant to high temperature and have effective quenching mechanisms and high photosynthetic capacity [58,59]. These physiological properties also might explain the significant negative correlation between the abundance of the member of this family with the concentration of solids in the influent. In addition, *Coleastrum* seemed to prefer more alkaline environment than *Tetradesmus* as it grew mainly in the PBR, where the pH was always higher than 8. *Chlorella* spp. exhibited a broad range of niche traits. *Chlorella sorokiniana*, rather than *Chorella vulgaris*, was favored in environments with high TAN, nitrate, and low irradiance, probably because of its more effective photoautotrophic specific growth rates [60]. On the contrary, *Chlamydomonas* sp. and *Micractinium* sp., exhibited higher tolerance toward nitrite. Contamination of *Gastrostyla* occurred in the RWP during the parallel worsening of the meteorological conditions and the increment of the concentration of COD. Such predators coexisted mainly with *Chlorella* sp., confirming that Chlorellaceae are more sensitive to grazing than *Scenedesmus* spp., for which colony formation and the presence of a spine provide a morphological defense against grazers [61]. 

As regards the cyanobacterial genera, *Calothrix* in the RWP was favored by high temperature as it has optimal growth rate at temperatures between 25 and 35 °C [62], as well as in low concentration of TAN being able to N_2_-fixation [63]. On the contrary, species belonging to the genera *Oscillochloris* and *Caldilinea* preferred an environment with low turbidity and low concentration of phosphorus. Several bacterial genera, such as *Kaistia, Plesiomonas*, *Denitrobacter*, *Rhodoplanes* and *Wautersiella*, were affected by the concentration of COD in the influent and the level of nitrate in the suspension, being heterotrophs and/or involved in the denitrification process. 

All these findings were gathered to delineate the plausible mechanisms (biotic and abiotic) driving the microbial population shifts in the reactors (Table 4). The table highlights how the continuous changes in abiotic conditions and resource availability perturbed the ecosystems, allowing the growth of microalgae and bacteria with more favorable niche traits. Nevertheless, several population changes could not be explained, suggesting a strong role of the stochastic factors in the microbial community assembly [11,12].

### 3.6. Network Analyses

To untangle the relationship between the bacterial and eukaryotic communities, a bipartite network for each reactor type was constructed (Table 5 and Figure S4). Overall, the RWP had a larger number of interactions than the PBR (see number of interactions, i.e., the number of connected elements, Table 5), though the latter showed a higher complexity of the interactions given the higher network density and heterogeneity. The network heterogeneity is shown by the presence of hubs within the network, while network density is given by the average edges per node, so that a network with many isolated nodes will have a density closer to zero. Communities that are highly connected and have high modularity (i.e., a clustered structured as in the PBR reactor) could be more functionally unstable, since the loss of a node has a higher effect on them [64]. The bloom of predators has a significant impact on the community structure, as predators mutually exclude with almost every other eukaryote as they are microalgae grazers. In general, the PBR experienced less contamination than the RWP over the entire experiment, but once predators entered the reactor they led to a complete disruption of the eukaryotic community. Such fragility can be probably associated to the highly clustered structure of the community [64]. On the contrary, the RWP harbored constantly non-microalgae eukaryotes in the community and the entry of new contaminants was unable to produce drastic damages to the community like in the PBR. In this case, the high diversity and dynamics observed in the RWP (Table 3 and Table 4) might help in establishing a complex interactive community (Table 5, interactions number and connected elements) that contrasted the spread of predators over time.

## 4. Discussion

### 4.1. Environmental Parameters, Biomass Productivity and Nutrients Removal in the Photobioreactors

The biomass productivity and overall nutrient removal were comparable in the two photobioreactors, though influenced by the environmental conditions. Both systems reached a total ammoniacal nitrogen and PO_4_^3−^-P removal rates of 21 and 0.45 mg L^−1^ d^−1^, respectively, which are in line with similar systems treating digestate located in the same geographical area. Low COD removal rate could be observed in the PBR and RWP, demonstrating that centrate was mainly composed of recalcitrant and not easily biodegradable compounds. This suggests that this type of wastewater is suitable for preventing the contamination of heterotrophic microbes (i.e., bacteria, fungi and protozoa) in outdoor microalgae cultivation. The microalgal growth and productivity (average 25 mg L^−1^ d^−1^) followed the variation of the temperature and light, which were typical of the area, and was impacted by erroneous operational strategies. The sudden drop in the microalgal biomass between day 60 and 73 in the RWP could be attributed to the coverage of the system with a shading net, which was placed to prevent photoinhibition. Meanwhile, the worsening of the meteorological conditions, such as low temperature and light at the end of the trial, led to a reduction in the microalgal growth. Overall, these finding suggest that, in suboptimal climate conditions, the performance of outdoor photobioreactors could be improved by the implementation of equipment to mitigate the irradiance and temperature variations, such as the use of external light and heating systems. Although the operational costs would increase, the system are expected to produce more biomass to valorize.

On the other hand, the biological nitrogen removal pathways in the systems were linked to the different configurations and operations of the reactors. Complete nitrification could be achieved only in the RWP, while partial nitrification occurred in the PBR. High ammonium oxidation is quite common in microalgae-based technologies treating municipal and agro-zootechnical digestate [34,54,55], especially if phosphorus is readily available [65]. The elevated P concentration in the centrate favored the activity of the AOB [66], which are the main responsible for ammonium oxidation. This hypothesis is supported also by the positive correlation between the ammonium oxidation rate and the concentration of PO_4_^3−^-P (PO_4_^3−^-P _out_ = 0.86 *pr*N-NO_x_ + 6.8, R² = 0.3623, *p*-value = 0.0038). Nevertheless, the number of detected AOB was lower than expected if considering the ammonium oxidation rate. It is plausible that a fraction of AOB, which prefer growing in colonies, often attached on cellular aggregate and/or within biofilm developing on the reactor walls or on the paddlewheel in the case of the raceway pond [54], were not sampled. Another plausible reason is that other microorganisms contributed to the process. For instance, *Pseudomonas veronii*, which was the dominant bacterial species, might have a role, as it possesses the *AmoA* gene encoding for the oxygen monooxygenase [67,68,69], and its function as heterotrophic nitrifier was already observed in natural and engineered ecosystems. However, more studies are required to confirm this hypothesis. 

Nitrite oxidation occurred in the RWP but it was seriously impaired in the PBR. Most likely, the higher pH of the column, shortage of CO_2_ and the slightly higher level of free ammonia than in the RWP prevented the NOB growth [28]. However, if complete nitrification is desired, the implementation of pH control systems and longer HRT might help in overcoming these issues.

### 4.2. Microbial Community Structure 

Species belonging to Scenedesmaceae and Chlorellaceae were the dominant microalgae observed in both systems, confirming their suitability for the bioremediation of digestate [8]. The non-axenic and open conditions of the bioreactors allowed for the entry of protozoa and bacteria, which actively interacted with the microalgae. As commonly observed in microalgal cultivation systems, predation, synergistic, antagonist and competitive mechanisms could be detected [13,70]. In the RWP, algivorous and microalgal grazers, belonging to *Ciliophora*, constantly co-existed with the microalgae, while in the PBR, predators were mainly detected when the microalgal growth was impaired. Nitrifiers and cyanobacteria, which are known to compete with the microalgae for CO_2_ and ammoniacal nitrogen, colonized both reactors to different extent. *Pseudomonas* sp., which are known to be plant growth-promoting bacteria [71], were probably beneficial for the microalgae by producing siderophores for iron absorption, solubilize phosphate and synthetize phytohormones, such as indole-3-acetic acid (IAA) [72,73]. On the other hand, antagonist interactions between bacteria and microalgae occurred, as demonstrated by the negative correlation between the number of bacteria and microalgal counts. This evidence contrasts with the frequently stated symbiotic relationship between microalgae and bacteria in microalgae-based technology treating wastewaters [70,74]. However, most of the synergetic interaction concerns the exchange of O_2_ and CO_2_ during the photosynthetic activity and the aerobic degradation of the organic matter by the heterotrophic bacteria. In our systems, most of the COD in the centrate was not easily biodegradable, as shown by the negligible COD removal rate. Therefore, the growth of the heterotrophs was fostered only when organic compounds were available by microalgal cells’ decay. 

The non-parallel evolution of the microbial populations in the two reactors indicates that the configuration and operational parameters had a key role in regulating the composition and dynamics of the microalgal and bacterial communities. Without a doubt, high irradiance and temperature were responsible for the shifts between Chlorellaceae and Scenedesmaceae in both reactors due to the more advantageous physiological traits of the latter in such conditions [58,59], but the diverse pH and concentrations of N compounds in each reactor promoted the selection of specific populations. In the RWP, the high level of nitrate, which was derived by complete nitrification, and the concentration of ammonium in the influent seems to favor *C. sorokiniana, P. nurekis* and their predator *G. steinii*, as well as denitrifiers. On the contrary, in the PBR, nitrite accumulation caused by the incomplete nitrification, promoted the growth of *Chlamydomonas* spp. and *Micractinium* sp., while the alkaline pH supported the establishment of members of *Chloroflexi*. Although not all the population shifts could be explained by the variations of the abiotic conditions because of the influence of stochastic factors [12], the association found between the microbes displaying specific functional traits and the environmental conditions established in each reactor might help to predict which populations are favored under various environmental and operational scenarios.

The communities in the RWP were more dynamic than those in the PBR, as demonstrated by the higher number of significant population shifts observed in both microalgal and bacterial communities. Nevertheless, the PBR was more subject to drastic events, while the RWP seemed to be more resilient to perturbations. In the RWP, the negative effect of the shading net on the microalgal composition could be barely detected, while a drastic increase in non-microalgal populations could be observed in the PBR. Yet, the microalgal community in the RWP recovered when the climate conditions got worse, but not in the PBR. The continuous perturbations of the ecosystems due to changes in the abiotic conditions and resource availability favored the growth of microalgae and bacteria with more favorable niche traits and migration of new microbes in both reactors. Nevertheless, the RWP constantly harbored non-algal eukaryotes suggesting more trophic interactions; the coexistence of predators and microalgae was more evident in the RWP than in the PBR, as well as predator diversity was higher in RWP than PBR (only one in PBR and three in RWP). It seems that the more complex community established in the RWP helped in contrasting the spread of the predators over time by maintaining the predator/prey dynamics under control, even if the raceway system has a wider open area and a design more prone to external contamination.

The establishment of a diversified microalgal community derived by the variable abiotic conditions seemed to play a role in maintaining the productivity of the reactors in agreement with other aquatic ecosystems [75,76] and microalgal cultivation systems [25,77]. The trends of microalgal density showed two peaks in both reactors. In the PBR, the highest and first peak corresponded to day 18 and 31, when the community was dominated by *Chlorella*. The extremely high temperature and irradiance of the following days led to a switch to the Scenedesmaceae, but a drastic decrement of the biomass. Biomass concentration and diversity increased again only with the mitigation of the climatic factors. Similarly, in the RWP, the initial biomass productivity was sustained by the Chlorellaceae and Scenedesmaceae; nevertheless, the highest concentration of biomass could be detected when the climatic and operational conditions were more variable. These findings endorse the use of microalgal polycultures for a more stable production of biomass in outdoor cultivation systems, especially if located in regions with a temperate climate where the environmental conditions are subject to seasonal variations 

## Figures and Tables

**Table 1:** Average physicochemical characteristics of the diluted centrate, which was used to feed the column (PBR) and in the raceway pond (RWP). Significant differences between reactors are also evidenced (*) (Paired -*t* test, *p*-value < 0.05).

	PBR	RWP	*p*-value
**COD (mg L^−1^)**	298 ± 48.7	376 ± 111	*
**NH_4_^+^-N (mg L^−1^)**	260.1 ± 61.9	288.2 ± 86.7	
**NO_3_^-^-N (mg L^−1^)**	7.9 ± 3.1	11.3 ± 5.1	
**PO_4_^3-^-P (mg L^−1^)**	14.6 ± 4	15 ± 9.8	

**Table 2:** Average chemical-physical parameters in column (PBR) and in the raceway pond (RWP), as well as the Chemical Oxygen Demand (COD) and nutrient removal rate observed. Significant differences between reactors are also expressed (*) (Paired -*t* test, *p*-value < 0.05).

	PBR	RWP	*p*-value
**pH**	8.5 ± 0.5	7.2 ± 0.6	*
**T_r_^§^**	24.5± 6.2	21.9 ± 7.2	*
***rr*COD (mg L^−1^ d ^−1^)**	−4 ± 10.7	5.7 ± 6.1	
***rr*TAN (mg L^−1^ d ^−1^)**	21.9 ± 7.3	19.5 ± 6.6	
***rp*NO_x_-N (mg L^−1^ d ^−1^)**	14.4 ± 12.1	18.1 ± 6.9	
***rp*NO_2_^-^-N (mg L^−1^ d ^−1^)**	13.5 ± 11.2	1.4 ± 2.2	*
***rp*NO_3_^-^-N (mg L^−1^ d ^−1^)**	1 ± 1.3	16.7 ± 7.9	*
**Free Ammonia (mg L^−1^)**	5.7 ± 8.5	1.8 ± 3.3	
***η*TAN (%)**	84.1 ± 13.1	79 ± 14.8	
***rr*PO_4_^3−^-P (mg L^−1^ d ^−1^)**	0.6 ± 0.9	0.3 ± 0.9	
***rp*VSS (mg L^−1^ d ^−1^)**	27 ± 20.3	23 ± 20.3	

^§^ T_r_ is the temperature detected inside the reactors.

**Table 3:** Summary of the total number of Eukaryotic (non-microalgae and microalgae) operational taxonomic units (OTUs) with a percentage above 1%, number of shared OTUs between the column (PBR) and the raceway pond (RWP), and number of OTUs detected in the sole PBR or RWP.

	Total	Shared	Only PBR	Only RWP
**Eukaryotes**	63	10	18	35
**Non-microalgal**	40	2	11	27
**Microalgae**	23	8	7	8

**Table 4:** Plausible mechanisms of the evolution of the microbial populations in the column (PBR) and the race way pond (RWP). Codes are the same as those reported in Figure 5 and Figure 6, while in brackets the time when the shifts occurred are indicated.

Eukariotes			
Code	PBR	Code	RWP
**Inoculum**	✓ Coexistence of *C. vulgaris* and *T. dimorphus*	**Inoculum**	✓ Coexistence of *C. vulgaris* and *T. dimorphus*
**PBR_Ek_1** **(31–45)**	✓ *Scenedesmaceae* (*T. dimorphus* and *C. proboscideum*) outcompeted *C. vulgaris*. Increase T and I	**RWP_Ek_1** **(18–31)**	✓ Bloom of *Vorticella microstoma* Undefined perturbation
**PBR_Ek_2** **(60–87)**	✓ Increase abundance of yeast and *Vorticella microstoma*. The reactor was covered with a shading net that killed the microalgae	**RWP_Ek_2** **(31–45)**	✓ Dominance of *T. dimorphus* Increase T and I
**PBR_Ek_3** **(87–115)**	✓ Most of the populations present before the crashed recovered; increase abundance of *Chlamydomonas* sp. and *Chlorella* sp. High NO_2_^-^-N; decrease I and T	**RWP_Ek_3** **(60–73)**	✓ *T. dimorphus* co-existed with *C. vulgaris* and *C. proboscideum*. Presence of predators. Mitigation of I and T conditions
**PBR_Ek_4** **(129–143)**	✓ Increased abundance of *Chlorella* sp. and *Micractinium* sp. and *Vorticella microstoma* High level of NO_2_^-^-N together with the decrease in NH_4_^+^-N and I	**RWP_Ek_4** **(87–115)**	✓ *C. sorokiniana*, *P. nurekis* and their predator *G. steinii*. Drastic increase in NH_4_^+^-N, NO_3_^-^-N and COD, together with the decrease in I and T
**PBR_Ek_5** **(143–171)**	✓ Dominance of *Vorticella microstoma* Worsening of the climatic conditions reduced the microalgal growth favoring predation	**RWP_Ek_5** **(115–129)**	✓ Increase *Chlorella* sp. and *Vorticella microstoma* Decrease influent NH_4_^+^-N and NO_3_^-^-N
		**RWP_Ek_6** **(143–171)**	✓ Increase number of predators The change of dilution of the centrate disturbed the microalgal community
		**RWP_Ek_7** **(171–188)**	✓ Microalgal community recovered after the system failure due to predation
****Bacteria****			
**Code**	**PBR**	**Code**	**RWP**
**Inoculum**	✓ Bacterial community of the inoculum	**Inoculum**	✓ Bacterial community of the inoculum
**PBR_Bac_1** **(0_18)**	✓ Establishment of a mixed community with *Pseudomonas* spp. as dominant bacteria	**RWP_Bac_1** **(0_18)**	✓ Establishment of a mixed community with *Pseudomonas* spp. as dominant bacteria
		**RWP_Bac_2** **(18_31)**	✓ Coexistence of *Pseudomonas* and *Calothrix* spp. Undefined perturbation decreased the number of microalgae; increase in I
		**RWP_Bac_3** **(31_45)**	✓ Dominance of Cyanobacteria.Further increased of I and T
		**RWP_Bac_4** **(60_73)**	✓ Recovery of the original communityMitigation of meteorological conditions
		**RWP_Bac_5** **(87_115)**	✓ Increase abundance of denitrifiers, such as *Kaistia* and *Rhodoplanes* Increase NO_3_^-^-N and COD in the influent, together with the reduction of T
		**RWP_Bac_6** **(143_188)**	✓ Recovery of the previous community

**Table 5:** Properties of bipartite networks based on the interaction between the most abundant (>1%) bacterial and eukaryotic OTUs.

Network Properties	PBR	RWP
Modularity	0.415	0.508
Connected component	101	117
Average degree	9.373	8.048
Average path length	1.982	2.314
Network centralization	0.2078	0.167
Network heterogeneity	0.7014	0.590
Number nodes	110	125
Network diameter	6	7
Network density	0.086	0.065
Interaction	2480	2588
Co-occurrence	1492	1488
Mutual exclusion	988	1100

## Supplementary Materials

The following are available online at https://www.mdpi.com/2076-2607/8/11/1754/s1, Figure S1: Images of the main microalgae, protozoa and cyanobacteria observed in the PBR and RWP over time; Figure S2: Number of the total eukariotic (N_EK_) (A) and bacterial species (N_BAC_) (B) detected in the column (PBR) and raceway pond (RWP) over time; Figure S3: Non-metric multidimensional scaling analyses of the eukaryotic (A and B) and bacterial OTU patterns (C and D) in the PBR (blue) and in the RWP (black). Numbers indicate the sampling date, while arrows represent directions of forcing of each environmental parameter measured in the influent (IN) and reactors (OUT). Torb is the turbidity. T_PBR_ and T_RWP_ is the temperature in the bubble column and in the raceway pond, respectively. I is the irradiance; Figure S4: Bipartite network based on the interaction between the most abundant (>1%) eukaryotic and bacterial OTUs in bubble columns and raceways pond (A and B).
