# Supplementary material for: Comparison of the Performance and Microbial Community Structure of Two Outdoor Pilot-Scale Photobioreactors Treating Digestate
Source: Microorganisms. 2020 Nov 8;8(11):1754. doi: 10.3390/microorganisms8111754 (PMC7695279; doi:10.3390/microorganisms8111754)
Supplement: Supplementary file 1 [file microorganisms-08-01754-s001.pdf]

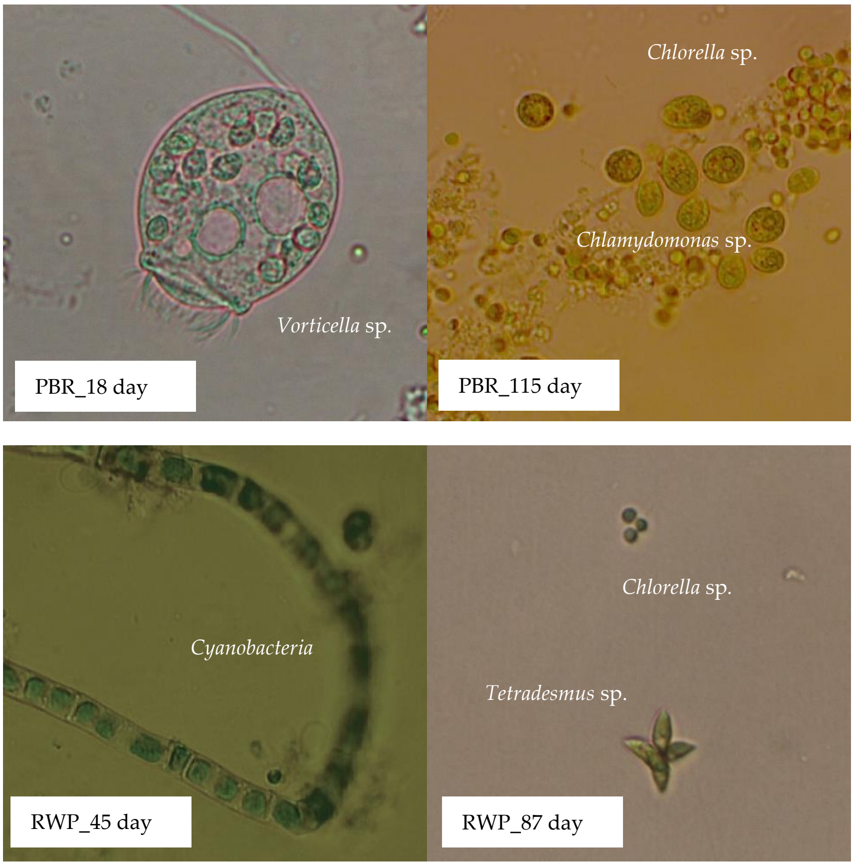

Figure S1. Images of the main microalgae, protozoa and cyanobacteria observed in the PBR and RWP over time.

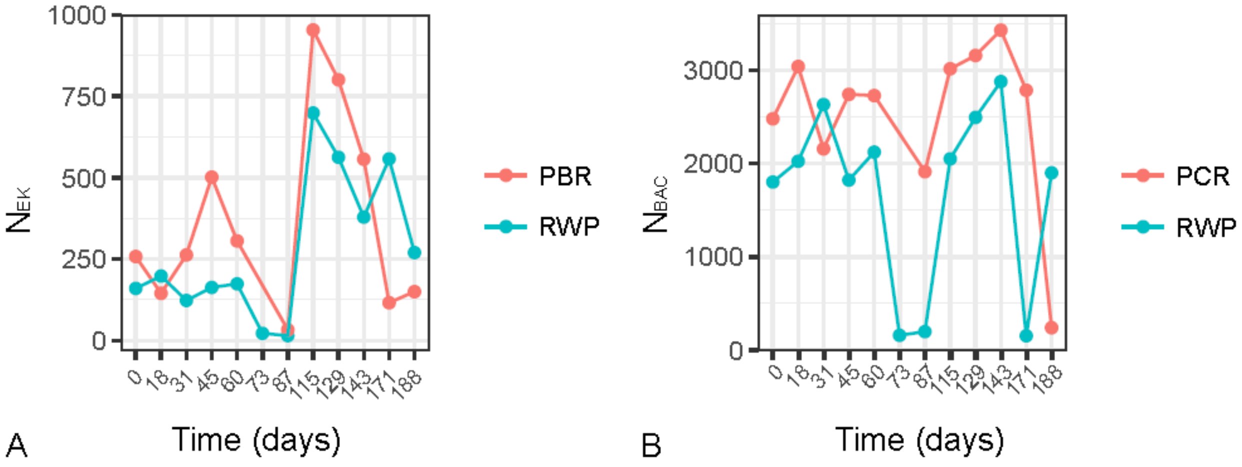

Figure S2: Number of the tolal eukariotic (N_{EK}) (A) and bacterial species (N_{BAC}) (B) detected in the column (PBR) and raceway pond (RWP) over time.

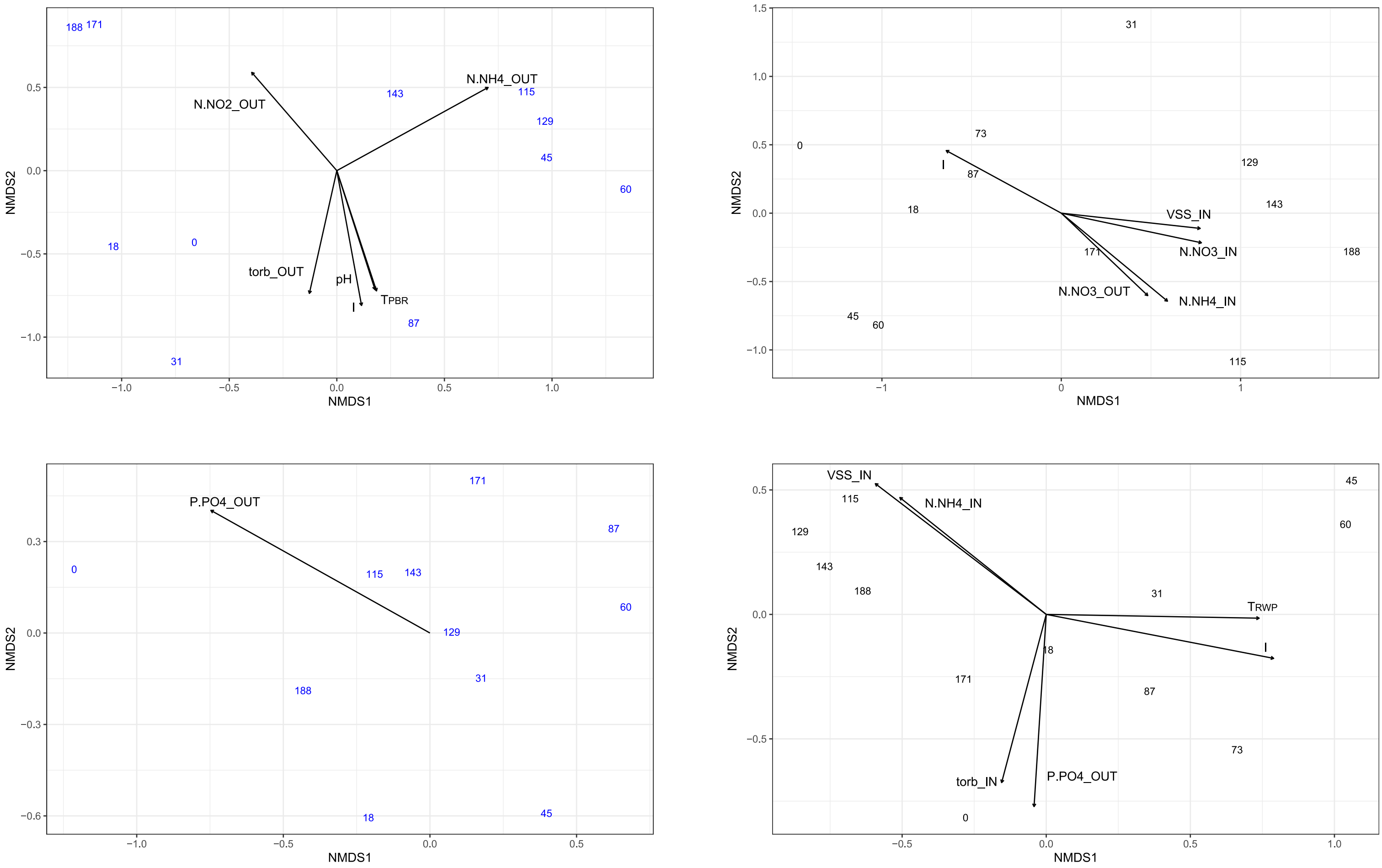

Figure S3: Non-metric multidimensional scaling analyses of the eukaryotic (A and B) and bacterial OTU patterns (C and D) in the PBR (blue) and in the RWP (black). Numbers indicate the sampling date, while arrows represent directions of forcing of each environmental parameter measured in the influent (IN) and reactors (OUT). Torb is the turbidity. TPBR and TRWP is the temperature in the bubble column and in the raceway pond, respectively. I is the irradiance.

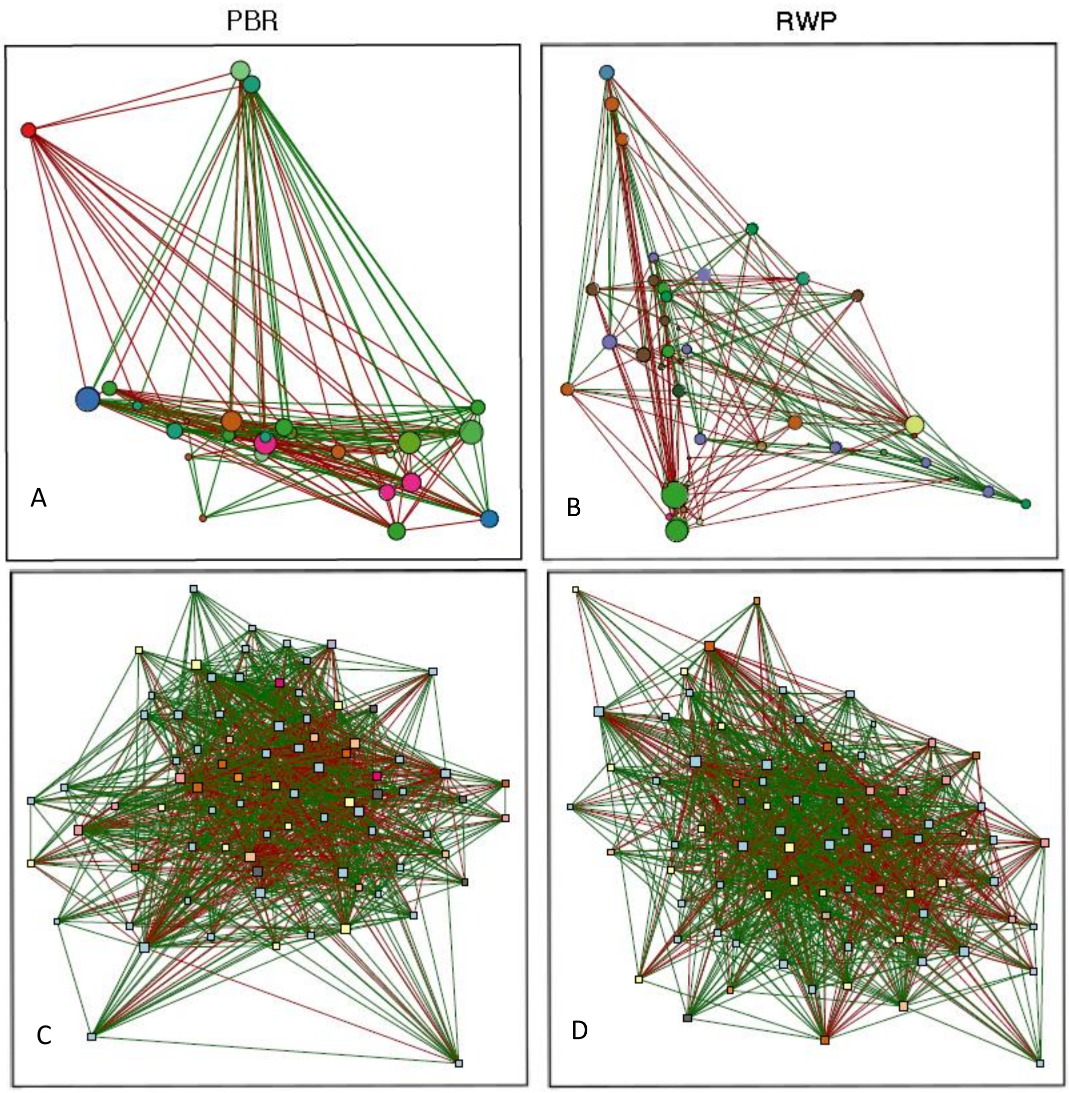

***Figure S4*: Bipartite network based on the interaction between the most abundant (>1%) eukaryotic (A and B) and bacterial (C and D) OTUs in the bubble columns (A and C) and raceway pond (B and D).
